# Automated generation of epilepsy surgery resection masks: The RAMPS pipeline

**DOI:** 10.1162/IMAG.a.147

**Published:** 2025-09-10

**Authors:** Callum Simpson, Gerard Hall, John S. Duncan, Yujiang Wang, Peter N. Taylor

**Affiliations:** CNNP Lab (www.cnnp-lab.com), Interdisciplinary Computing and Complex BioSystems Group, School of Computing, Newcastle University, Newcastle upon Tyne, United Kingdom; UCL Queen Square Institute of Neurology, Queen Square, London, United Kingdom; Faculty of Medical Sciences, Newcastle University, Newcastle upon Tyne, United Kingdom

**Keywords:** epilepsy, surgery, resection, volume, mask

## Abstract

MRI-based delineation of brain tissue removed by epilepsy surgery can be challenging due to post-operative brain shift. In consequence, most studies use manual approaches which are prohibitively time-consuming for large sample sizes, require expertise, and can be prone to errors. We propose RAMPS (Resections And Masks in Preoperative Space), an automated pipeline to generate a 3D resection mask of pre-operative tissue. Our pipeline leverages existing software including FreeSurfer, SynthStrip, Sythnseg and ANTs to generate a mask in the same space as the patient’s pre-operative T1 weighted MRI. We compare our automated masks against manually drawn masks and two other existing pipelines (Epic-CHOP and ResectVol). Comparing to manual masks (N = 87), RAMPS achieved a median (IQR) dice similarity of 0.86 (0.078) in temporal lobe resections, and 0.72 (0.32) in extratemporal resections. In comparison to other pipelines, RAMPS had higher dice similarities (N = 62) (RAMPS: 0.86, Epic-CHOP: 0.72, ResectVol: 0.72). We release a user-friendly, easy-to-use pipeline, RAMPS, open source for accurate delineation of resected tissue.

## 1 Introduction

Surgical removal of the epileptic foci as a treatment for drug-resistant epilepsy leads to seizure freedom in only 50% of cases (De Tisi et al., 2011). This fact sparked a wave of retrospective studies focused on re-examining the preoperative tissue under new hypotheses (Ahmed et al., 2015; Galovic et al., 2019; Hall et al., 2023; Horsley et al., 2023; Keller et al., 2015; Sainburg & Morgan, 2024; Taylor et al., 2018), all aiming to uncover novel biomarkers to predict surgical outcome. In all such studies, accurate delineation of the resected tissue is crucial to capture precise information.

A common approach to delineate the resection is manually drawing by hand a resection mask, a 3D binary matrix labeling individual T1-weighted voxels as a part of the resection cavity (Taylor et al., 2018). Manual delineation is time-consuming, requiring a skilled individual with advanced neuroanatomical and surgical knowledge (Arnold et al., 2022; Gau et al., 2020). Even with such knowledge, the manually generated mask varies between raters (Pérez-García et al., 2020).

These issues highlight the need for an automated technique capable of delineating the resection cavity to 1) reduce time investment, 2) reduce domain knowledge needed, increasing the accessibility to resection mask-based analysis, 3) enable comparison of results against additional resection classifications, and 4) facilitate easier replication of previous studies on new datasets.

**Fig. 1.**
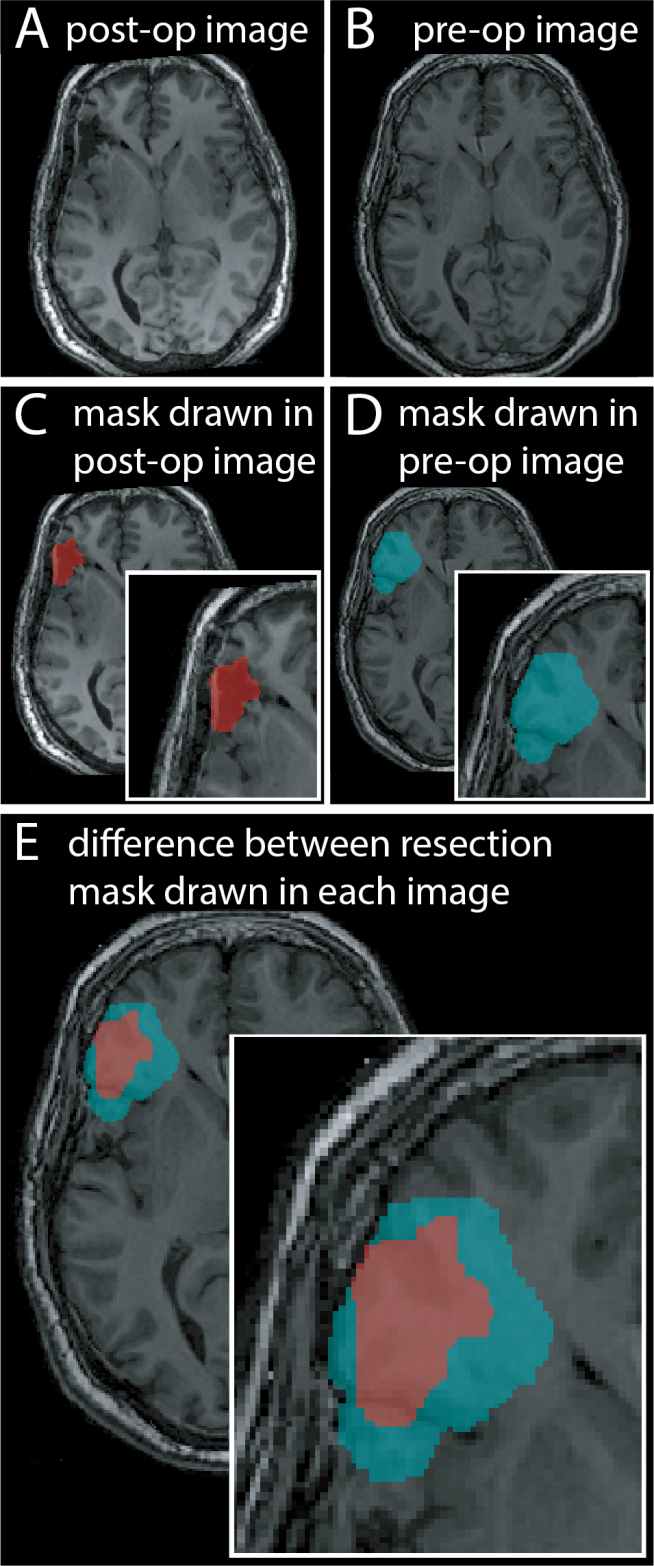
The need to generate resection masks in preoperative space. Illustrated is the axial aligned (A) post-operative and (B) the pre-operative T1w images for a frontal lobe resection. Following surgery, sagging is noted within the resected cavity. (C) shows a manually drawn mask following the resection cavity seen in the post-operative image. (D) shows a manually drawn resection mask that indicates the pre-operative tissue that is shown to be resected in the post-operative image. (E) Highlights the difference between these two resection cavity interpretations.

Several tools to delineate the resection cavity exist in three categories; deep/machine learning (Arnold et al., 2022; Casseb et al., 2024; Pérez-García et al., 2021), semi-automated (Billardello et al., 2022; Wilke et al., 2011) and fully automated statistical (Cahill et al., 2019; Casseb et al., 2021). Though pipelines differ methodologically, all aim to output a resection mask. Many of these resources generate the mask in the post-operative space by filling in the resection cavity (Fig. 1C). These approaches yield good results when compared to other manual masks also drawn in the post-operative space, with median dice similarities in the range of 0.74 to 0.82 (Arnold et al., 2022; Pérez-García et al., 2021). However these approaches are prone to errors caused by post-operative sagging, swelling, or brain shift into the resection cavity. Visual comparison of the post-operative mask to a mask delineating the pre-operative tissue resection (Fig. 1D) highlights differences in volume and extent (Fig. 1E). This problem can be further exacerbated if the registration between the two images is poor, causing additional warping to the resection mask. The creation of resection masks in pre-operative, rather than post-operative space is therefore of paramount importance.

Here, we present the RAMPS pipeline (Resections And Masks in Pre-operative Space). This pipeline generates masks of the subsequent resection in pre-operative space. Using a three-part pipeline building on previously available tools, we automated the creation of pre-operative resection masks, highlighting the pre-operative tissue shown to be resected in the post-operative image. This is achieved using a patient’s pre-operative and post-operative T1w image, information regarding resected lobe and hemisphere information. RAMPS is open access, and all code is freely available to download (https://github.com/cnnp-lab/RAMPS).

## 2 Materials and Methods

### 2.1 Data used

In this study, we used pre- and post-operative T1-weighted MRI brain scans along with lobe and hemispheric information about the site of surgery from 87 individuals who had epilepsy surgery. In each case, a manual resection mask was drawn on the pre-operative scan, highlighting the tissue shown to be resected in the post-operative scan. To validate the performance under diverse conditions, there were no exclusion criteria when selecting patients. Scans include Temporal Lobe Epilepsy TLE (N = 70) and Extra Temporal Lobe Epilepsy ETLE cases (N = 17), collected from different centers, and 3D T1-weighted images were acquired across different protocols. The majority of the scans were acquired at the Chalfont Centre, with manual masks delineated for previous studies (Hall et al., 2023; Taylor et al., 2018), with additional scans being collected from the University Hospital Iowa (N = 2 used by Kocsis et al. (2023)), Pennsylvania, and Mayo Clinic. Information about the patient cohorts (Supplementary Table S1) and the images used (Supplementary Table S2) can be found in the Supplementary Materials.

### 2.2 Manual segmentation

All patients had a manually drawn mask delineating the pre-operative tissue that was subsequently resected. This was achieved by visually comparing the post-operative and pre-operative scans against one another to emphasize what tissue was resected then slice by slice highlighting the pre-operative voxels that adhere to the resection and saving the output as a binary image. These masks were created by 3 individuals trained in this process. The time and effort required to manually segment a resection varied based on size of resection and warping caused by swelling/sagging. The majority (>95%) of the manual masks were created using the FMRIB Software Library (FSL) image viewer FSLeyes, or FSLview (Jenkinson et al., 2012). For this study, all manual masks were filtered by a binary segmentation of the pre-operative brain tissue to remove erroneous voxels outside the brain (e.g., CSF, bone). If not already in 1 mm ^3^ resolution, manual masks were converted to match this resolution.

### 2.3 RAMPS pipeline

**Fig. 2.**
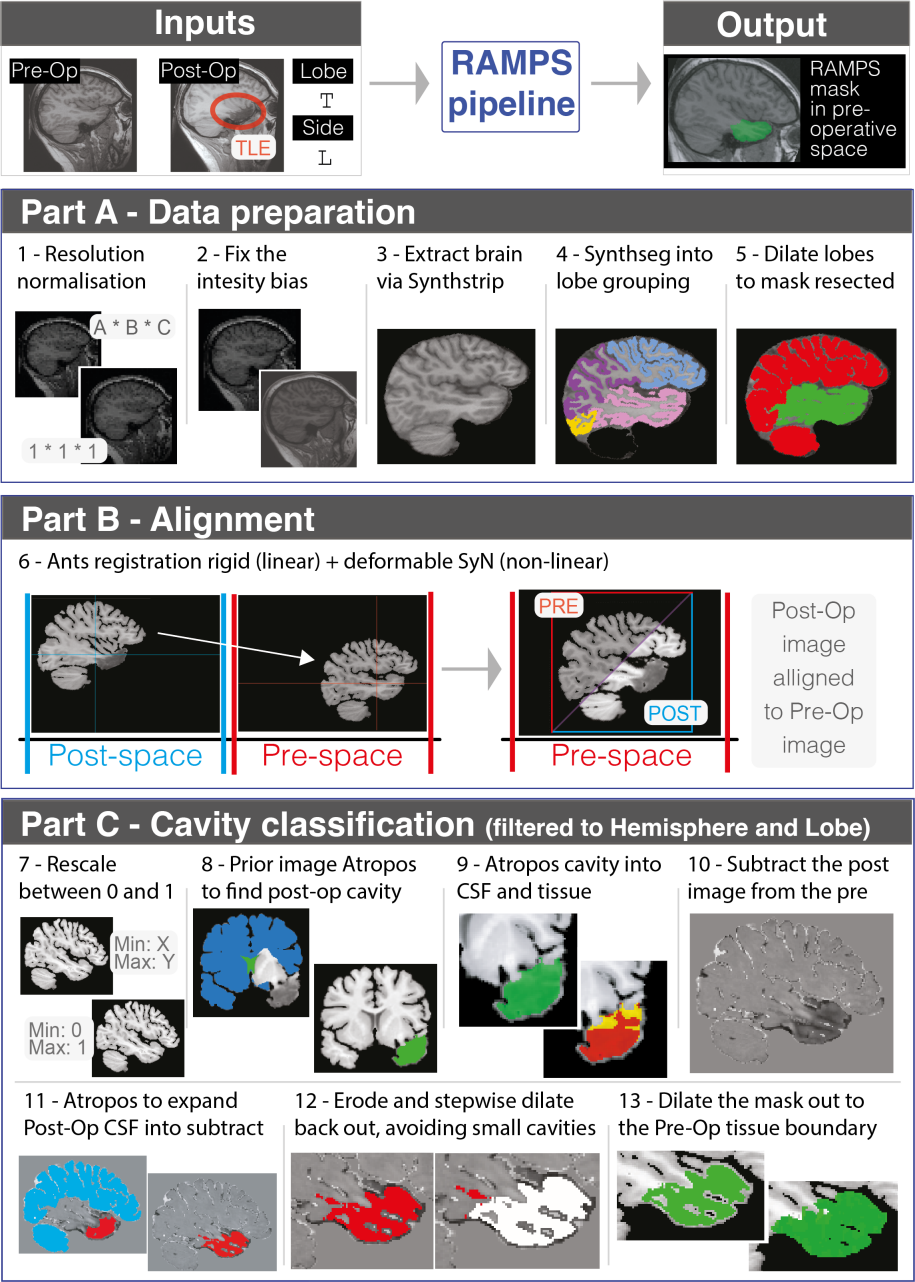
Overview of the RAMPS pipeline. Using a T1w pre- and post-operative image, as well as hemisphere and lobe of resection, RAMPS generates a mask of resected pre-operative tissue in 3 steps. Step 1 is data preparation, in which a series of steps are undertaken to remove noise from the image, extract the brain tissue, and create a lobe atlas map of the brain. Step 2 uses ANTs registration to align the post-operative brain to the pre-operative. Step 3 is mask creation which involves delineating the resection cavity in the post-operative space resection and then expanding to the pre-operative tissue boundary.

The RAMPS pipeline comprises 3 core parts: data preparation, registration, and mask creation (Fig. 2). RAMPS runs in Python and uses modules such as nibabel (Brett et al., 2024) and ANTs (Tustison et al., 2021) to aid mask creation.

#### 2.3.1 Required parameters

RAMPS requires the following:

- Pre-operative image—A T1w image captured before surgery.
- Post-operative image—A T1w image captured after surgery, with a clear surgery.
- Output folder—The folder where the outputs of RAMPS will be stored.

While the code can be run with just this information, we recommend providing:

- A subject ID.
- Hemisphere initials where the resection took place (L, R, or both). Default is both meaning RAMPS will search both hemispheres for a resection cavity.
- Lobe of resection initial(s), if the resection spans multiple lobes then multiple can be specified. (T, F, O, P). Temporal lobe filter also includes Sub-cortical and insula regions. Default is all lobes.

Hemisphere and lobe inputs to ensure that a mask is generated in the correct area and is assumed as prior knowledge from the end-user.

#### 2.3.2 Part A: Data preparation

To enhance image alignment and improve the ease of creating a resection mask, there are a series of data preparation steps to remove noise and generate lobe classifications masks.

- **Step 1—Resolution normalization.** First apply the ANTs (Tustison et al., 2021) resample_image_to_target function to resample images into 1 x 1 x 1 mm resolution with 265 × 256 × 256 fov. This keeps the images in their original space while standardizing FOV and voxel size.
- **Step 2—Bias intensity correction.** Images are then run through *ants.n4_bias_field_correction* (Tustison et al., 2010) to correct any low-frequency intensity non-uniformity present in MRI image data known as a bias or gain field. This corrects the slow and smooth intensity variation across the image, thereby reducing field bias. At the end of part A, to further ensure bias removal, the top 1% voxels values were replaced by the median voxel value.
- **Step 3—Brain extraction.** Removal of non-brain elements (i.e., skull, eyes, etc.) reduces potential complications in the segmentation and registration step, especially around the resection cavity (Casseb et al., 2021). The skull-stripping tool SynthStrip is used remove non-brain elements from the T1w images. SynthStrip has been shown to be a robust model and agnostic to acquisition specifics (Hoopes et al., 2022).
- **Step 4—Regional segmentation.** Through the use of SynthSeg (Billot et al., 2023), the images are segmented into a series of atlas regions. SynthSeg is robust across various brain scans of differing contrast and resolution. Atlas regions are then joined via lobe to create a grey-matter lobe atlas map of the following regional categories: Frontal, Parietal, Temporal, Occipital, Insula, Sub-Cortical areas and areas in which the resection cannot occur (such as ventricles, brainstem, and cerebellum). Additionally, the SynthStrip brain image is multiplied by a binarized mask made from the SynthSeg segmented atlas. The rationale is to eliminate any remaining non-brain elements or residual surface left in the image, particularly around the resection cavity.
- **Step 5—Lobe of resected area.** The grey-matter lobe atlas map is dilated throughout the white matter to create a full lobe map. The dilated atlas is subsequently divided into two binary masks based on users’ specification: the hemisphere-specific lobe where the resection occurred, and the other lobes where the resection did not occur.

#### 2.3.3 Part B: Post-operative image alignment into pre-operative image space registration

- **Step 6—Registration.** At this stage, the T1w images have been cleaned but still exist in different coordinate spaces. Before creating the resection mask, align the tissue in the post-operative image to the pre-operative image. Due to the sagging and swelling that may be seen in post-operative image, this alignment is critical, as poor alignment could erroneously cause further misalignment of tissue. The ANTs antsRegistrationSyN rigid + deformable syn approach was found to be accurate in the presence of sagging and swelling. In addition, the calculated transformations are used to move information created from the post-operative image into the pre-operative space.

#### 2.3.4 Part C: Cavity classification

Following registration, the post-operative image exists in the pre-operative space and the following steps produce a resection mask in pre-operative space.

- **Step 7—Rescale.** To contrast the pre- and post-operative images against one another, first rescale the images between 0 and 1, to ensure that the cerebrospinal fluid (CSF), grey matter, and white matter exhibit similar voxel values across images.
- **Step 8—Post-operative image atropos.** Next, we delineate the resection cavity within the post-operative image. This is achieved through ANTs Atropos (Avants et al., 2011), an open-source finite mixture modeling algorithm for tissue segmentation. Here, we use ANTs with Prior Label Image initialization, a clustering technique that groups voxels based on a prior segmentation of each class. The prior images used here consist of 1) ventricles for classification of cerebrospinal fluid (CSF) and 2) cerebral tissue in the non-resected lobes. This classifies the voxels in the resected lobe into 1) the resection cavity and 2) non-resected tissue.
- **Step 9—Post-operative image find cavity CSF.** The previous step classifies the image based on similarities to the prior voxel intensities, however in-between voxels corresponding to damaged tissue may be included in the resection cavity. To separate the damaged tissue from CSF, a no prior image two group K-mean Atropos classification was applied within the step-8 resection cavity. As no voxel priors are utilized, the cluster with the lowest median voxel intensity is classed as CSF.
- **Step 10—Image subtraction.** Next, subtract the post-operative rescaled image from the pre-operative rescaled image to create a difference image. This approach is based on the rationale that after step 7, subtraction of the same voxel class (grey matter, white matter, and CSF) will roughly equal zero, whereas overlap of differing classes will be not equal zero. This highlights the overlap between the resection cavity observed in the post-operative image (CSF) and tissue in the pre-operative image, and indicates where sagging has occurred within the image. The resulting subtraction image is then multiplied by the mask of pre-operative image, highlighting the areas of tissue difference in the pre-operative image.
- **Step 11—Atropos through subtraction image.** Similar to step 8, Atropos is used with a prior image to expand the post-op resection cavity through the subtraction image. This highlights the voxels in the subtracted images where the images differ. Additionally, the results of this Atropos are filtered to the lobe in which resection takes place and the largest object is selected to be the mask of the resection cavity.
- **Step 12—Cavity removal.** A common issue with the subtraction image arises from poor registration, leading to differences caused by tissue misalignment and not resection. These sections of poor alignment are often attached to the main resection mask but via narrow contact points of a few voxels. These areas of noise can be removed by first eroding the mask and performing a series of small dilations through the original mask. After each dilation, we examine the voxels expanded into. If expansion reveals a small cluster of voxels, it indicates that there will be an area of poor alignment. Further dilation into this region is prevented, re-creating the step 11 mask, but removing these areas of misalignment.
- **Step 13—Boundary dilation.** To ensure that the mask extends to the appropriate tissue boundary, a directional dilation is performed. If a given voxel on the boundary of the mask is within 3 voxels of CSF, all voxels between the two points onto the mask are added. This stops the dilation into tissue that was not resected.
- **Step 14—Additional cleaning.** ANTs morphology is applied to fill any small cavities within the resection mask. Then, the mask is multiplied by a binarized mask of the pre-operative lobe of resection to remove any voxels that might exist outside this region. Finally, the resection mask is resampled back into the pre-operative resolution.

### 2.4 Data analysis

The primary analysis evaluated the masks produced via RAMPS with lobar and hemispheric information against the manually drawn cohort. First, the dice similarity coefficient (DSC) between the RAMPS and manual masks across the TLE and ETLE cohort was computed. Additionally, the miss rate/false discovery rate of the RAMPS mask produced to highlight how close they are to a manually drawn mask was examined. The secondary analysis compared the results of RAMPS to those from additional automation tools (Epic-CHOP and ResectVol) using both DSC and miss rate/false discovery rate approaches to evaluate the effectiveness of RAMPS.

### 2.5 Statistical analysis

As in other studies (Arnold et al., 2022; Casseb et al., 2021, 2024; Courtney et al., 2024; Wilke et al., 2011), we compared the RAMPS resection mask to manually drawn masks using the Dice Similarity Coefficient (DSC). The DSC has shown to be a robust metric of both overlap and reproducibility, ranging from 0 (no overlap) to 1 (which in this study indicates a 1 to 1 replication of the manually drawn mask) (Fyllingen et al., 2016). DCS between 0 to 0.6 is poor, 0.6 to 0.7 is good, 0.7 to 0.8 is considered high, and values exceeding 0.8 are excellent (Seghier et al., 2008). We calculated the median DSC and the interquartile range (IQR).

Additionally, the overlap of the two masks was viewed as a confusion matrix to extract additional metrics for analysis, and to provide a more complete representation of alignment. The voxels which feature in the two masks are true positives, voxels that only exist in the manual are false negatives, voxels that only exist in the automated masks are false positives, and voxels that are outside both masks are true negatives. Using these definitions, we can calculate the miss rate, which quantifies the percentage of resected tissue not included by the automated mask and false discovery rate (FDR), a percentage measure of the automated mask that included extra non-resected tissue. The optimal result would be 100% overlap with 0% in miss rate and FDR, and this indicates a perfect overlap between masks.

### 2.6 Comparison to other pipelines

The effectiveness of RAMPS was compared with two other state-of-the-art tools that aim to generate a resection mask in the pre-operative space, Epilepsy Cavity Characterisation Pipeline (Epic-CHOP) (Cahill et al., 2019) and ResectVol (Casseb et al., 2021). Both of these pipelines find the resection cavity using Statistical Parametric Mapping (SPM12) software (Friston et al., 1994) and operate within MATLAB. Both tools follow a similar approach to mask creation, aligning the pre- and post-operative images and then finding the differences between the two. While the RAMPS process is similar, the three pipelines use different registration techniques, vary in their operational sequences, and employ different methods to generate the final resection mask.

ResectVol and Epic-CHOP were executed using the default values, though as no PET images exists for these subjects Epic-CHOP was filtered to only non-PET steps. By default, Epic-CHOP produces a series of possible resection masks. We evaluate the largest cluster mask produced which underwent all cleaning steps. While the possibility exists that other generated masks could more accurately delineate the resection cavity, it has been shown that Epic-CHOP provides no difference between the median DSCs achieved when using the largest or the most optimal representation mask (Courtney et al., 2024). Automated masks were converted to 1 mm^3^ resolution and filtered by a binary segmentation of the pre-operative brain tissue to keep the masks within the tissue to match the manual mask.

Epic-CHOP and ResectVol were performed on the same cohort of 87 subjects’ pre- and post-operative scans; however, some cases could not be processed by the additional pipelines with their default set up, producing hard errors on computation. Thus, we compared the output in the 62 subjects whose data were usable for all pipelines across the metrics of DSC, overlap, miss rate, and FDR. Additionally, RAMPS without lobar and hemispheric information was tested to evaluate how RAMPS performs when given only the MRI images. To examine the statistical differences between each pipeline, we used Wilcoxon signed-rank tests using the R function wilcox.test (paired = TRUE), a nonparametric, paired statistical test with the null hypothesis that the results achieved from differing pipelines are similar. One-tailed tests were conducted in both directions to assess whether one pipeline outperformed the other across each metric, with the ‘greater’ alternative applied to DSC and overlap, and the ‘less’ alternative applied to miss rate and false discovery (FDR). Significance was defined as p < 0.05.

### 2.7 Ethics

The studies involving human participants were reviewed and approved by Newcastle University Research Office Ref: 1804/2020.

## 3 Results

### 3.1 RAMPS performance using the dice similarity coefficient (DSC)

**Fig. 3.**
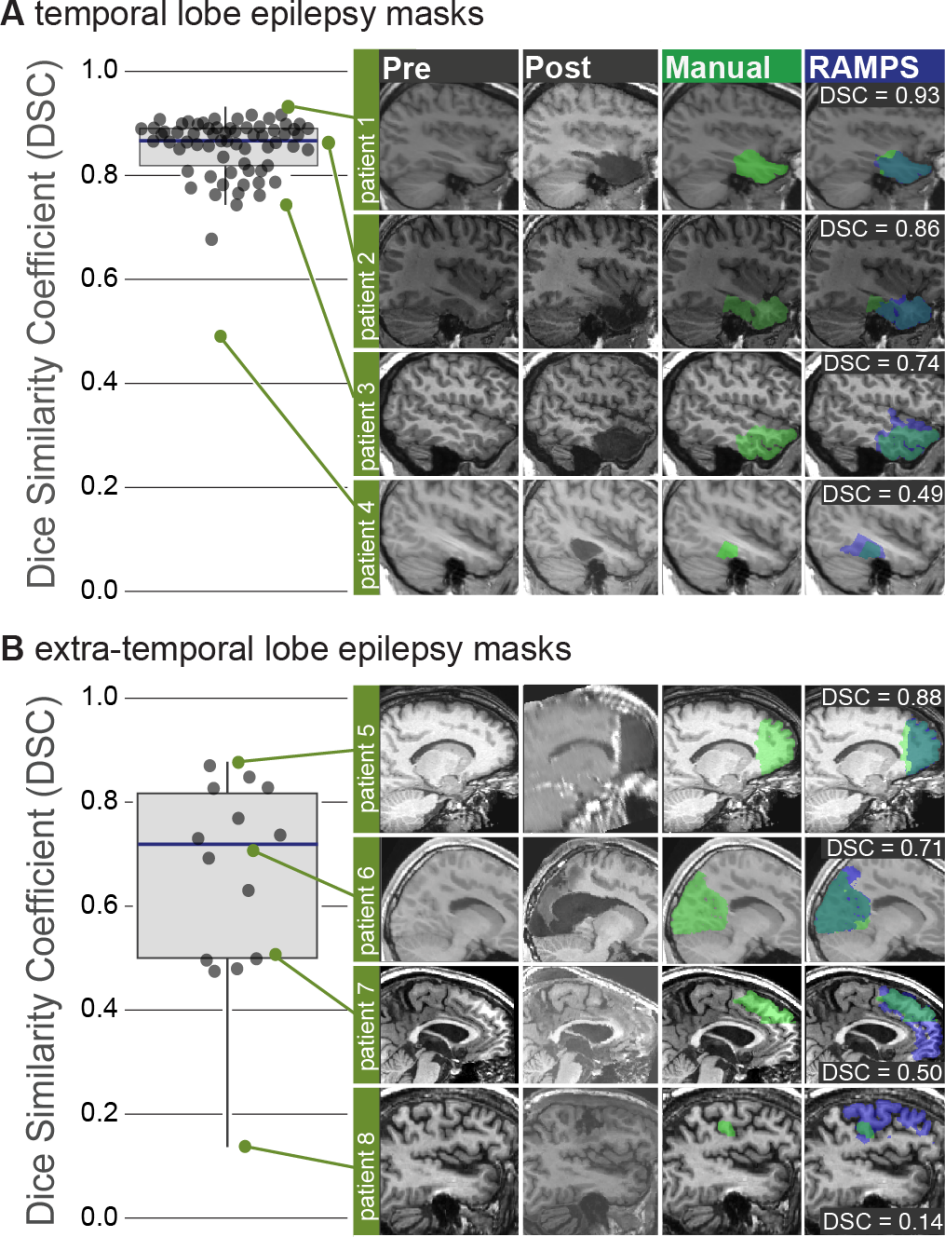
Similarity of manual and RAMPS generated masks. Using the Dice similarity coefficient for mask comparison, the TLE cohort (A) achieved a median similarity of 0.86 (IQR, 0.078) and the ETLE cohort (B) achieved a median similarity of 0.71 (IQR, 0.32). Example images, along with generated masks are shown in right panels.

The results from this primary analysis are presented with mask examples in Figure 3a for TLE and Figure 3b for ETLE. Comparing RAMPS given lobe and hemisphere information to manually drawn masks, in the TLE cohort there was an excellent median DSC of 0.86 (IQR, 0.078) and a high median DSC of 0.71 (IQR, 0.32) in the ETLE cohort. These results are similar to the inter-rater variability in pre-operative mask creation (Supplementary Fig. S1). One mask in the TLE group was poor (DSC < 0.6), as were 6 in the ETLE group. TLE had 68 cases (97%) and ETLE had 10 (53%) cases with high DSC (DSC >= 0.7).

### 3.2 RAMPS evaluation under different performance metrics

**Fig. 4.**
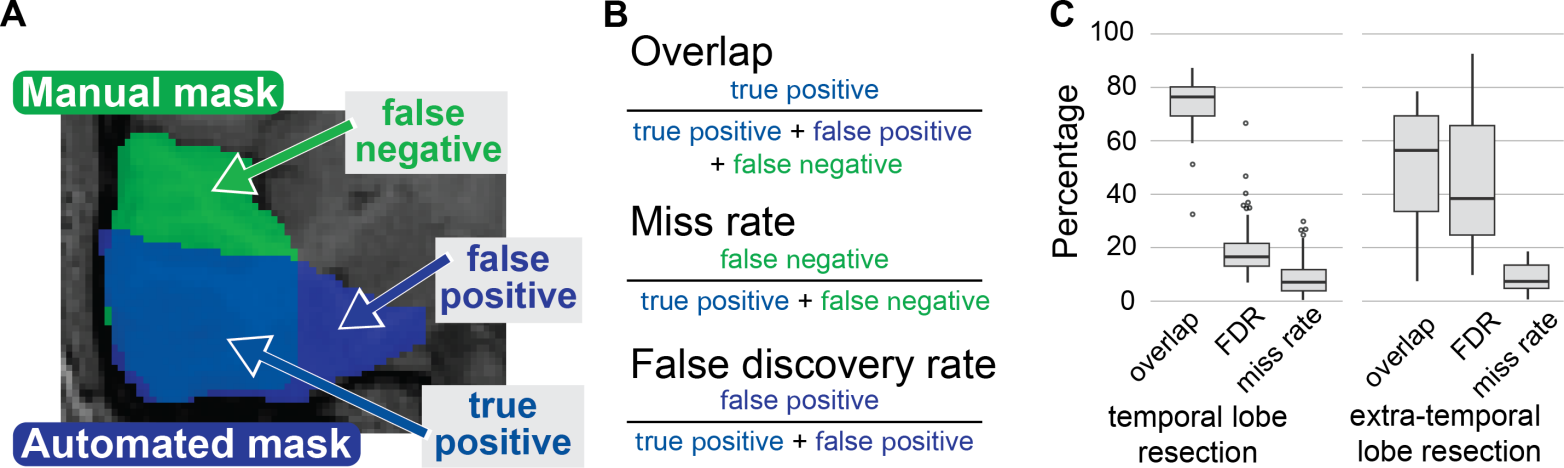
Performance metrics to analyze mask similarity besides DSC. (A) Example of a possible manual and an automated mask. This overlay can be divided into three categories: true positive, which are voxels included in both masks; false negatives, which are voxels included only in the manual mask; and false positives, which are voxels included only in the automated mask. (B) Using these metrics, the following may be calculated: overlap, which measures how effective the automation is at capturing the manual mask; miss rate, representing the percentage of the manual mask not captured; and False discovery rate (FDR), indicating the percentage of voxels identified by automation that fall outside the Manual mask. (C) Shows the results of the comparison of RAMPS to manual under these metrics.

Masks generated for TLE had a higher overlap (median 76% TLE, 55% ETLE), lower false discovery rate (median 16% TLE, 40% ETLE), and lower miss rate (median 7% TLE, 7% ETLE) than for ETLE (Fig. 4).

### 3.3 Comparison of different pipelines

**Fig. 5.**
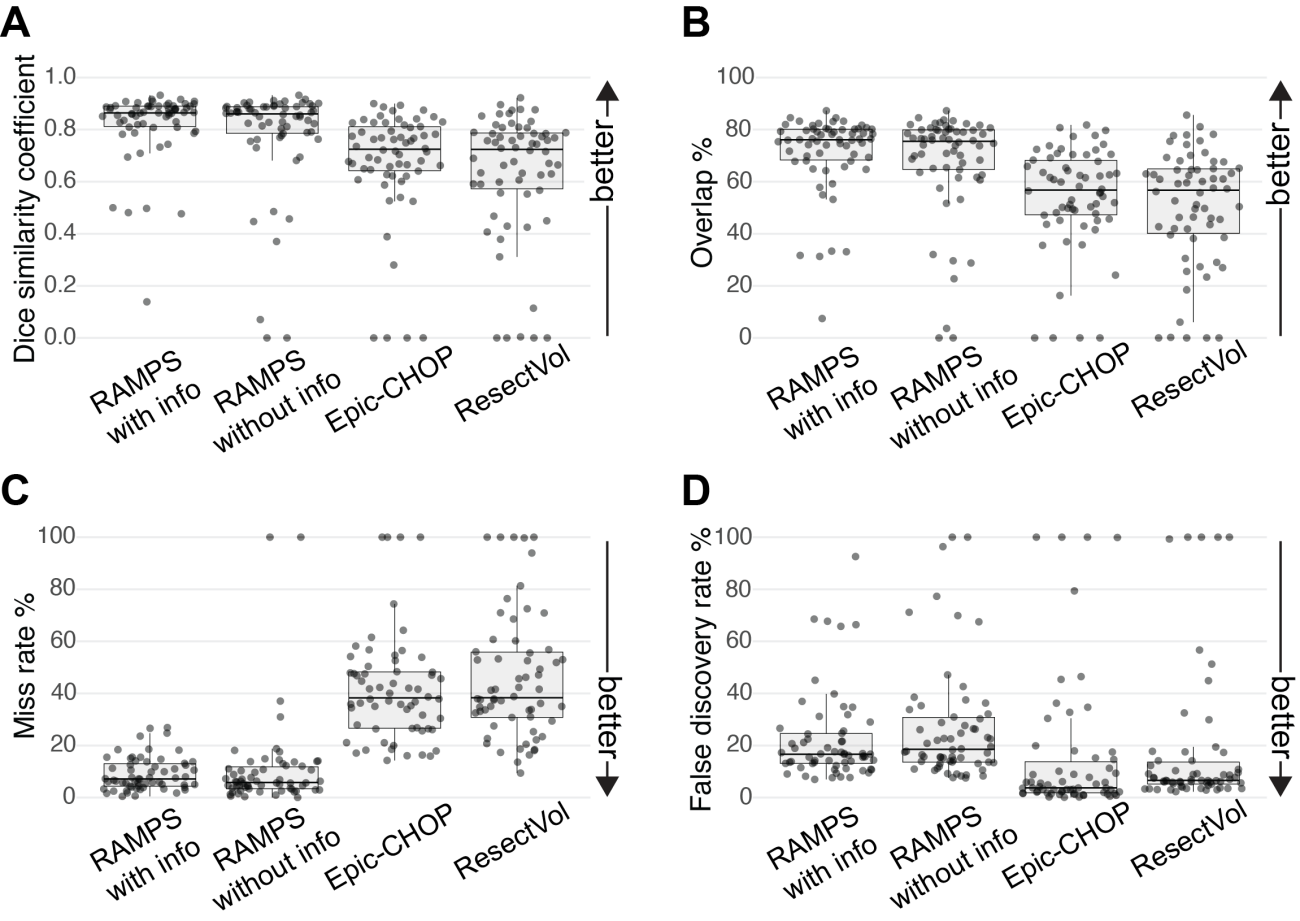
Pipeline comparison. The automated masks produced by RAMPS with lobe and hemisphere information, RAMPS without this optional information, and Epic-CHOP and ResectVol compared against a cohort of manual masks (N = 62). Each data point represents an individual patient. The median (IQR) were: (A) DSC metrics: RAMPS with 86% (7%), RAMPS without 86% (10%), Epic-CHOP 72% (16%), and ResectVol 72% (21%), (B) overlap metrics: RAMPS with 76% (11%), RAMPS without 75% (15%), Epic-CHOP 56% (21%), and ResectVol 56% (24%), (C) miss rate: RAMPS with 7% (9%), RAMPS without 6% (8%), Epic-CHOP 38% (21%), and ResectVol 38% (25%), (D) False discovery rate: RAMPS with 17% (12%), RAMPS without 18% (17%), Epic-CHOP 4% (12%), and ResectVol 7% (8%).

It was possible to process 62 subjects across the default set up of both additional pipelines (Fig. 5). RAMPS with lobar and hemispheric information achieved the highest DSC median (IQR) at 86% (7%), followed by RAMPS without at 86% (10%) with Epic-CHOP and ResectVol achieving 72% (16% and 21% respectively) (Fig. 5A). For median (IQR) overlap, RAMPS with had the highest at 76% (11%), RAMPS without achieved 75% (15%), Epic-CHOP achieved 56% (21%), and ResectVol achieved 56% (24%) (Fig. 5B). The optimal miss rate is zero and RAMPS without achieves the best median at 6% (8%), followed closely by RAMPS with 7% (9%) with both Epic-CHOP and ResectVol scoring 38% (21% and 25% respectively) (Fig. 5C). The optimal false discovery rate is 0, Epic-CHOP was best with a median (IQR) FDR at 4% (12%), ResectVol 7% (8%), RAMPS with at 17% (11%), and RAMPS without at 18% (17%) (Fig. 5D).

Both versions of RAMPS demonstrated significantly better performance in the DSC, miss rate, and overlap metrics compared to Epic-CHOP and ResectVol (all p < 0.001; Supplementary Table S3). RAMPS with lobar and hemispheric information performed significantly better than RAMPS without in DSC, overlap, and FDR (all p < 0.001). However, RAMPS without achieved a significantly better miss rate than RAMPS with (p < 0.001). For FDR, Both Epic-CHOP and ResectVol significantly outperformed both version of RAMPS (all p < 0.001). Across all metrics, Epic-CHOP achieved significantly better results than ResectVol (all p < 0.05).

**Fig. 6.**
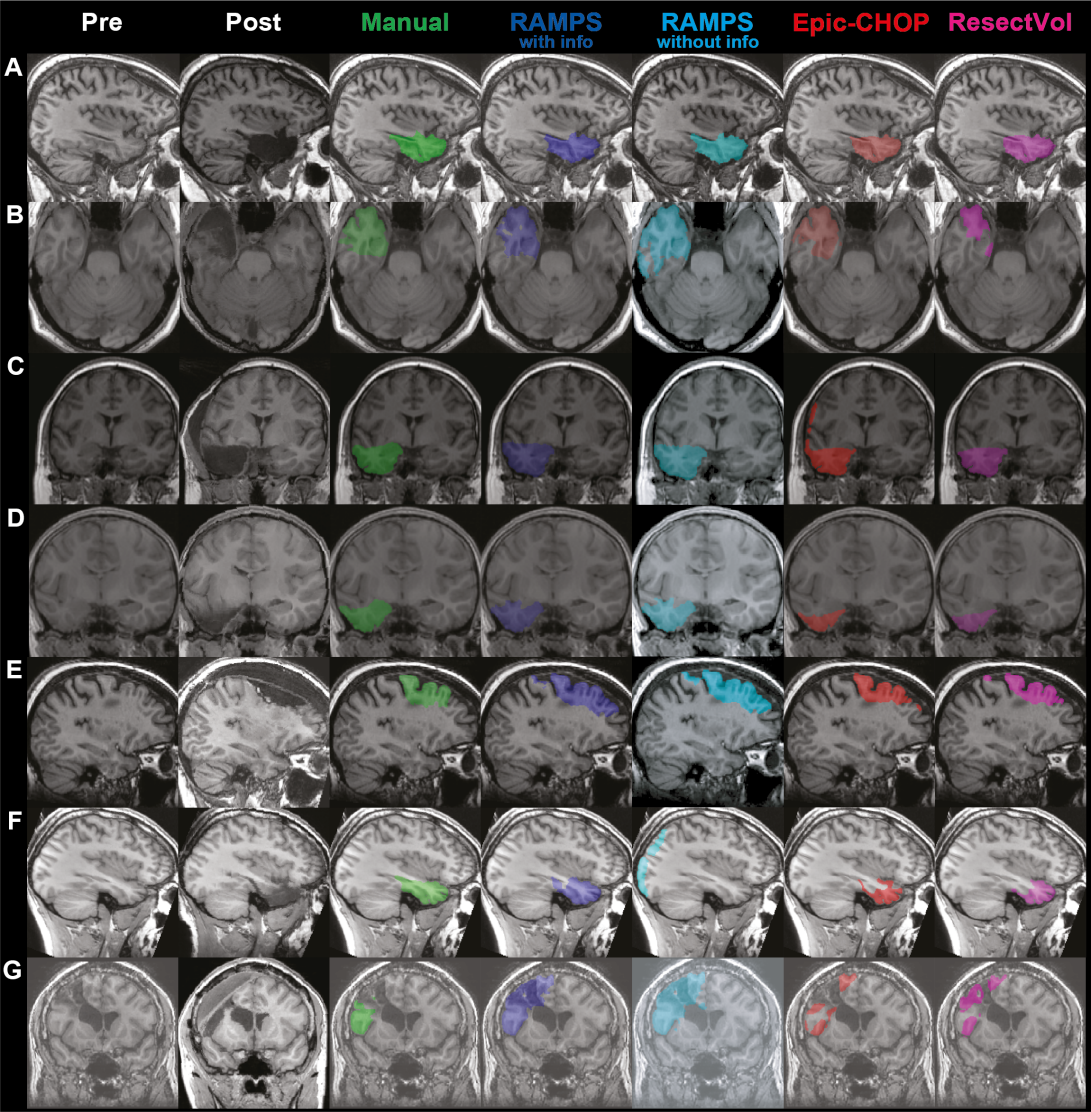
Visualization of resection mask output. The Pre and Post-operative images with the corresponding manually drawn mask and the automated resections masks produced RAMPS with lobe and hemisphere information, RAMPS without this optional information, Epic-CHOP and ResectVol to illustrate differing pipeline Dice similarity coefficient (DSC) performance across examples. (A) All pipelines yield high DSC (RAMPS with – 0.89, RAMPS without – 0.89, Epic-CHOP – 0.89, ResectVol – 0.86), (B) ResectVol yields lower DSC (RAMPS with – 0.82, RAMPS without – 0.77, Epic-CHOP – 0.82, ResectVol – 0.69), (C) Epic-CHOP yields lower DSC (RAMPS with – 0.88, RAMPS without – 0.87, Epic-CHOP – 0.71, ResectVol – 0.88), (D) RAMPS outperforms (RAMPS with – 0.88, RAMPS without – 0.87, Epic-CHOP – 0.62, ResectVol – 0.63), (E) RAMPS yields lower DSC (RAMPS with – 0.48, RAMPS without – 0.48, Epic-CHOP – 0.65, ResectVol – 0.59), (F) RAMPS with lobe and hemisphere information outperforms RAMPS without (RAMPS with – 0.85, RAMPS without – 0., Epic-CHOP – 0.65, ResectVol – 0.62), (G) All pipelines performed poorly (RAMPS with – 0.48, RAMPS without – 0.44, Epic-CHOP – 0.54, ResectVol – 0.45).

The inference from the miss rate and FDR is that the RAMPS masks produced by RAMPS are generally larger than those manually drawn, whereas Epic-CHOP and ResectVol are typically smaller and hence have fewer false discoveries. Visual comparison of the outputs produced by the pipelines alongside the images used to create them and the manual mask are shown in Figure 6.

### 3.4 RAMPS use with unusual cases

**Fig. 7.**
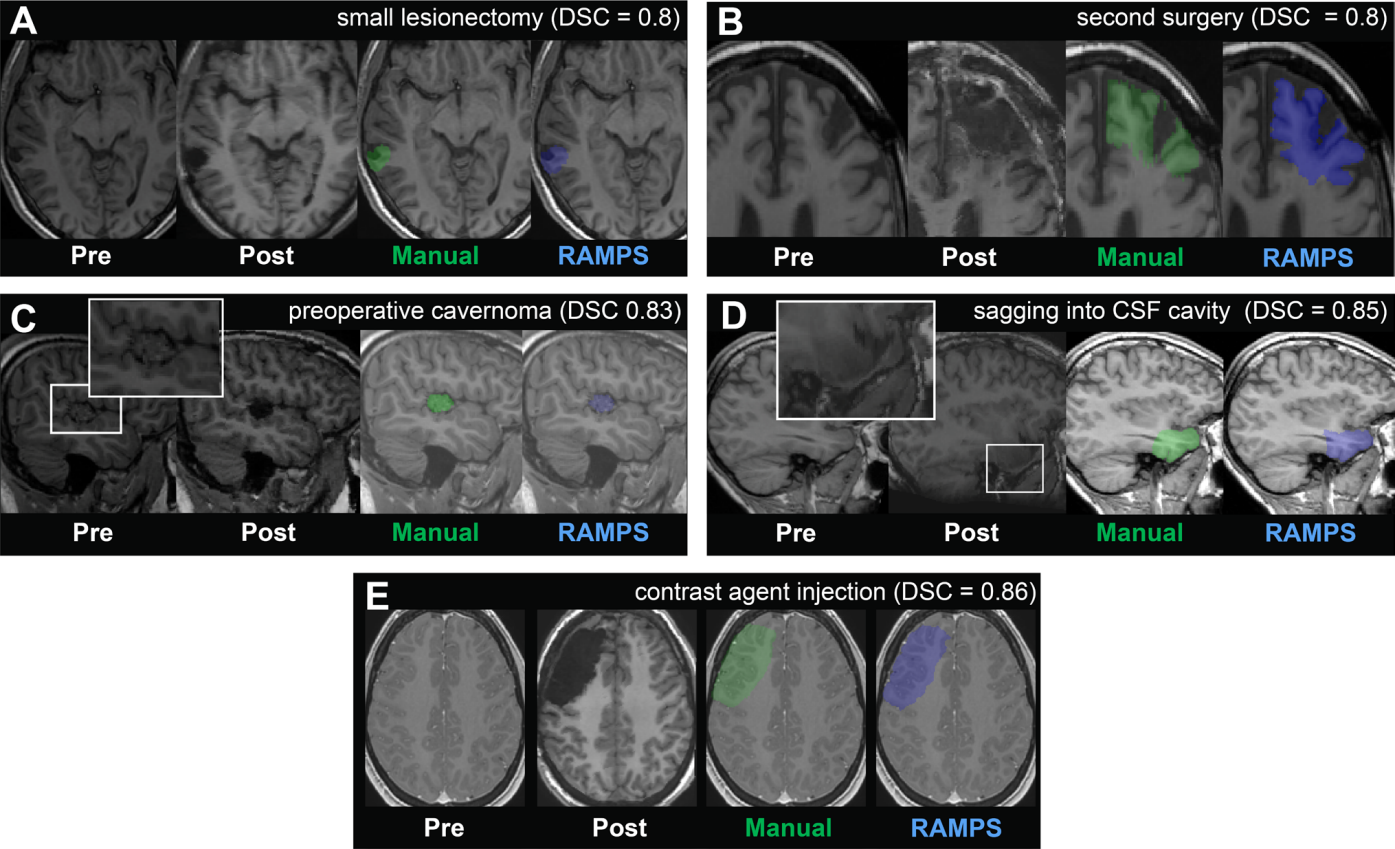
Demonstrating RAMPS’ DSC performance under nonstandard situations. Here, we visualize the automated masks produced for (A) a small lesionectomy (DSC = 0.8), (B) a second surgery (DSC = 0.8), (C) preoperative cavernoma (DSC = 0.83), (D) extreme sagging into the CSF cavity (DSC = 0.85), and (E) contrast agent injection (DSC = 0.85).

Finally, we evaluate RAMPS using lobe and hemisphere information performance in individual cases that differed from the main cohort, including a small lesionectomy, a second surgery, preoperative cavernoma, extreme sagging into the CSF cavity, and pre-operative contrast agent injection scan (Fig. 7). The DSC, when compared to a manually drawn mask, demonstrates excellent similarity in all cases (DSC >= 0.8), and further demonstrates RAMPS ability to perform segmentation across a diverse range of cases.

## 4 Discussion

Accurate delineation of resected tissue is of high importance in the study of epilepsy surgery. Most studies delineate tissue manually; however, this is costly in time and expertise and varies between raters. We present RAMPS, a fully automated tool for delineation of the pre-operative tissue that was found to be resected within the post-operative image. We compared RAMPS automated masks against 87 manually drawn masks gathered from a diverse cohort. We obtained excellent mask similarity with manual masks in TLE (median DSC = 0.86) and a high DSC in ETLE (median DSC = 0.71). As inter-rater variability is DSC 0.8 (Supplementary Fig. S1A), these results suggest RAMPS is able to segment to the level of a human rater.

The dice similarity coefficient is widely used in this type of analysis (Arnold et al., 2022; Casseb et al., 2024; Casseb et al., 2021; Courtney et al., 2024; Wilke et al., 2011). DSC exhibits bias toward larger volumes, as reflected by moderate positive spearmans correlation of 0.39 between Manual resection size and RAMPS DSC (Supplementary Fig. S4). Additionally, DSC does not inform *how* the masks are misaligned.

To overcome this limitation, we examined the percentage differences between the automated masks, in terms of overlap, miss rate, and false discovery rate. In the TLE cohort, RAMPS had a median (IQR) overlap of 76% (12%), a miss rate of 7% (8%), and an FDR of 16% (9). This highlights that RAMPS was able to capture 93% of the manual mask with misalignment primarily coming from over extension in some areas. In the ETLE cases, the overlap median was only 54% (37%); but, RAMPS still captured 90% of the manual masks volume. The use of additional metrics gave a more complete picture of the algorithm’s success.

RAMPS transformed the time-expensive task of resection cavity delineation to an automated computational process. This is beneficial when needing to analyze a cohort of images, and the mask creation can be sped up through additional computational resources. RAMPS is capable of generating masks on par with a manual rater and, as seen in Figure 3, RAMPS may classify the pre-operative tissue better than the manual rater in a few cases (Fig. 3, patient 4, 6, 8). Users, however, should still visually review the masks for accuracy. As indicated by the miss rate seen in Figure 4, a majority of the gold standard mask exists within the RAMPS mask so it may be quickly trimmed by human raters to match the manual evaluation of the resection.

Other studies have shown effectiveness of automatically generating a resection mask (Arnold et al., 2022; Casseb et al., 2024; Courtney et al., 2024; Wilke et al., 2011). However, only a few create the mask in pre-operative space. This generation space is crucial as most studies will analyze pre-operative data. Here, we compared two previously established pipelines with RAMPS: Epic-CHOP (Cahill et al., 2019) and ResectVol (Casseb et al., 2021). Additionally, as these pipelines operate without incorporating lobar and hemispheric information, RAMPS without this information was also ran for comparison. Both versions of RAMPS achieved significantly higher DSC, overlap and miss rate compared to ResectVol and Epic-CHOP (Supplementary Table S3). RAMPS given lobar and hemisphere information achieved significantly better DSC, overlap, and FDR scores compared to RAMPS without this information. ResectVol and Epic-CHOP achieved a significantly better FDR than both versions of RAMPS. However, both ResectVol and Epic-CHOP had a miss rate of 38%, which is higher than RAMPS FDR and miss rate combined, meaning more effort would be needed to bring these automated masks to the gold standard. Comparison between RAMPS using lobar and hemispheric information, ResectVol and Epic-CHOP on case-by-case basis can be observed in Supplementary Figure S2.

Visual comparison of pipeline output provides insight into why RAMPS outperforms other pipelines (Fig. 6). The ResectVol underperformance could be attributable to the generation of small volume masks (Fig. 6B and Supplementary Fig. S3), and Epic-CHOP is due to the introduction of noise along the image surface (Fig. 6C). We hypothesize that these deviations stem from misalignment between the pre- and post-operative images. Inadequate alignment around the resection cavity would fail to correct brain sagging/swelling, resulting in smaller masks (Fig. 6D). Additionally, as mask creation relies on differences between images, surface misalignment may erroneously be interpreted as a cavity. We credit RAMPS performance is due to the importance placed on image alignment and tailored post alignment cavity classification steps to ensure appropriate segmentation of pre-operative anatomy. RAMPS underperformance arises from classification overextensions when compared to the manually drawn masks (Fig. 6E). Comparing both versions of RAMPS, we see that without the lobe and hemisphere information, the automated mask produced may not delineate the resection cavity (Fig. 6F). Since RAMPS assesses the difference between the pre-operative and post-operative image, and the lobe and hemisphere information limits the search to a given location, discrepancies can arise when the largest cluster of voxel difference does not relate to the resection cavity (i.e., uncorrected intensities caused by low frequency bias that lie outside the lobe and hemisphere of resection).

Epic-CHOP and ResectVol automated masks were filtered within a binary brain mask, a step built into RAMPS, to ensure examination of delineation solely within the brain. Without this step, the other pipelines masks could include additional non-cerebral voxels, further increasing the false discovery rate and lowering DSC. Though there exist cases where no pipeline performed adequately (Fig. 6G), in some cases the other pipelines did not successfully produce a mask in the correct location (i.e., DSC = 0).

A limitation of this study is that we compared the automatically generated masks to only one manually drawn mask. Since there is variability between manual raters, there is no clear gold standard for comparison. Additionally, analysis metrics comparing the automated to manual masks assume the manual masks are entirely accurate. However, inadequate manual classification of the resected tissue would compromise analytical performance. Figure 3b patient 8 showcases this situation, where both the manual and automated resection mask fails to accurately delineate the resection cavity, leading to poor performance across the metrics, including FDR. Conversely, this suggests a potential advantage of automated approaches, as the automatically generated masks may, in some cases, be more accurate than the manual references used for comparison. Nonetheless, we strongly recommend visual inspection of all generated masks to ensure their reliability.

Most post-operative scans used in this study are from anterior temporal lobe resections (ATLR), the most common epilepsy surgery, in addition to a limited but diverse selection of ETLE scans. We observed performance differences between the TLE and ETLE cohort across all pipelines (Supplementary Fig. S5), as previously reported (Courtney et al., 2024). However, in terms of inter-rater variability, location of resection did not affect the median DSC variability between raters (Supplementary Fig. S1C—TLE 0.807 and Supplementary Fig. S1D—ETLE 0.815), suggesting differences are specific to automated approaches. This variation between cohorts may be due to reduced axis directions for variation. TLE resections are simpler to delineate because of clear anatomical boundaries of the temporal lobe, meaning variation in the resection primarily exists along the anterior-posterior axis, while any post-operative sagging occurs primarily in the superior-inferior axis. In contrast, ETLE resections vary in size and location so they are more affected by structural changes such as ventricular dilation. These factors lead to greater variations in delineations across the anterior-posterior, left-right and superior-inferior axis creating more opportunity for misalignment. While performance dropped in ETLE cases (Supplementary Fig. S5), both versions of RAMPS outperformed the other pipelines in terms of DSC, miss rate and overlap across the TLE and ETLE cohorts (Supplementary Table S4). RAMPS performance was significantly better in the TLE cohorts across these metrics (Supplementary Table S5), but in the ETLE cohort RAMPS only produced a significantly better miss rate (Supplementary Table S6). Additionally, in the ETLE cohort, RAMPS with lobe and hemisphere information produced significantly better DSC and overlap than ResectVol and RAMPS without, but the difference compared to Epic-CHOP was not significant.

Another potential limitation of RAMPS is the optional request for the user to input additional information regarding the hemisphere and lobe of resection for optimal usage, as other pipelines can operate without this request. As this information can be extracted through visual inspection, or from clinical notes, this is not expected to be a burden on users. This inclusion ensures RAMPS creates a mask in the correct tissue area, as shown with RAMPS capturing the cavity (DSC 0) in all cases. The RAMPS pipeline, however, can operate without this information while still achieving comparable results (Fig. 5), though performance decreased in a number of cases. Compared to the other pipelines, RAMPS without information still outperforms in DSC, overlap, and miss rate. Though both versions achieve excellent results, RAMPS run with lobar and hemispheric information significantly outperformed RAMPS run without this information in DSC and overlap (Supplementary Table S3). We recommend RAMPS be run with additional information to ensure capture of the resection cavity (Fig. 6F).

While we show RAMPS’s effectiveness in a diverse cohort, development depended on the data we had available. We would aim to extend the pipeline in future to include (i) Laser Interstitial Thermal Therapy (LiTT) ablations, (ii) disconnections, and (iii) alternative imaging besides T1w MRI, specifically post-operative CT. We release RAMPS as an open-source tool and encourage users to modify RAMPS to meet individuals situations and needs.

RAMPS is a new pipeline to generate masks of a resected cerebral cavity seen in the post-operative image, registered to the tissue resected in the pre-operative image. We provide RAMPS as a free, open-source, robust, easy-to-use tool, which we anticipate will be particularly useful for large datasets (Taylor et al., 2024).

## Supplementary Material

Supplementary Material

## Data Availability

Raw preoperative MRI scans are available as part of the IDEAS dataset (Taylor et al 2024). Code is available at the following location: https://github.com/cnnp-lab/RAMPS.
