# Supplementary material for: Automated generation of epilepsy surgery resection masks: The RAMPS pipeline
Source: Imaging Neurosci (Camb). 2025 Sep 10;3:IMAG.a.147. doi: 10.1162/IMAG.a.147 (PMC12423638; doi:10.1162/IMAG.a.147)
Supplement: Supplementary Material [file IMAG.a.147_supp.pdf]

Supplementary material.

Automated generation of epilepsy surgery resection masks; The RAMPS pipeline

Callum Simpson[1], Gerard Hall[1], John S. Duncan[3],
Yujiang Wang[1,2,3*], Peter N. Taylor[1,2,3],

1. CNNP Lab (www.cnnp-lab.com), Interdisciplinary Computing and Complex BioSystems Group, School of Computing, Newcastle University, Newcastle upon Tyne, United Kingdom
2. Faculty of Medical Sciences, Newcastle University, Newcastle upon Tyne, United Kingdom
3. UCL Queen Square Institute of Neurology, Queen Square, London, United Kingdom

* peter.taylor@newcastle.ac.uk

Supplementary

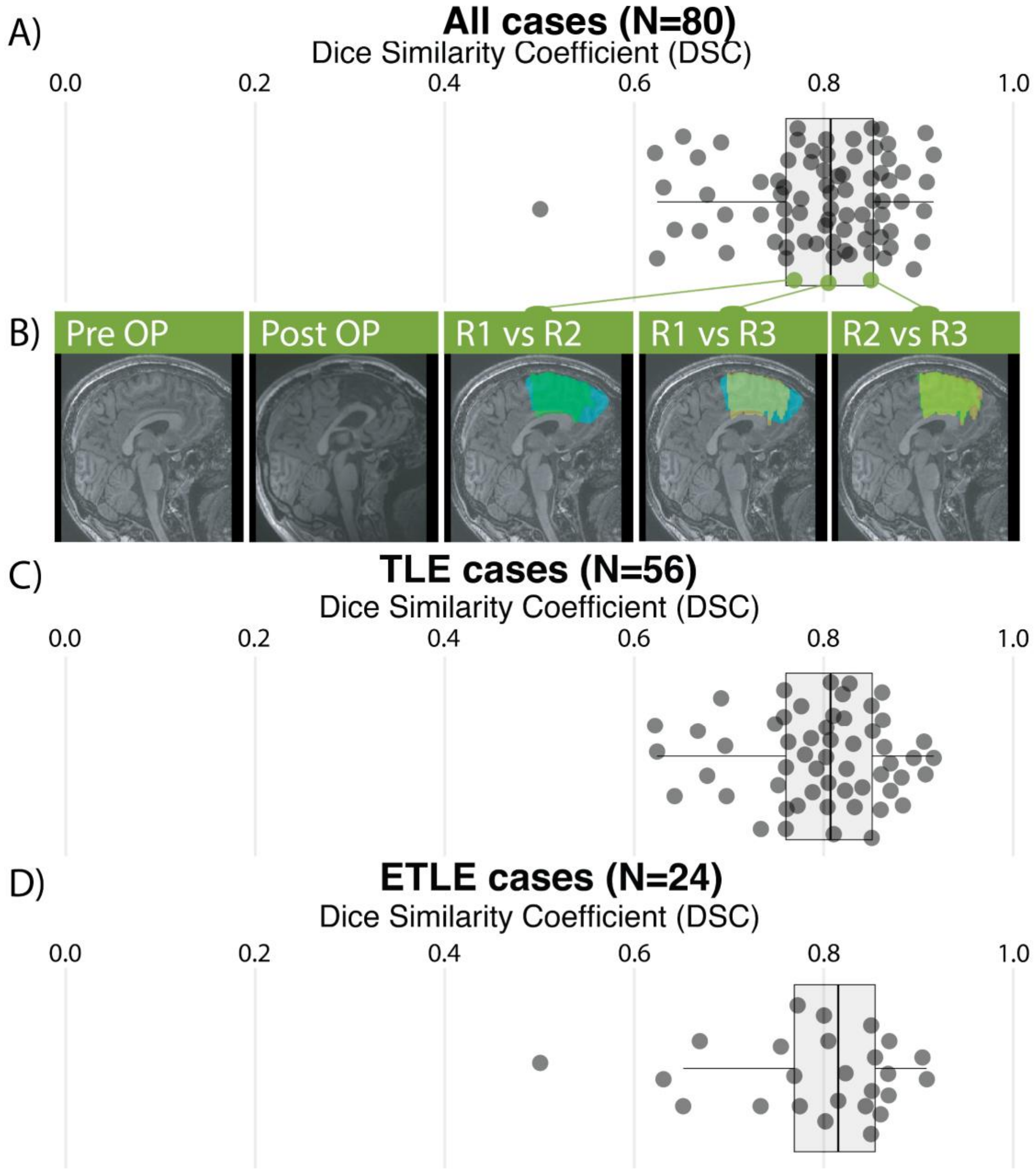

Figure S1: Inter-rater variability in manual resection mask creation. *To examine if differences between manual raters exists, the dice similarity coefficient (DSC) was calculated by comparing resection mask generated by two different raters segmenting the same cavity in pre-operative space. A) Analysis of 80 cases shows the median(IQR) DSC between two raters is 0.807(0.09). B) Illustrates a case where three raters segmented the same cavity, emphasizing how inter-rater variability can occur. C) In a subcohort of 56 TLE cases, the median(IQR) intra-rater DSC was 0.807(0.09). D) In a subcohort of 24 ETLE cases, the median (IQR) intra-rater DSC was 0.815(0.09)*

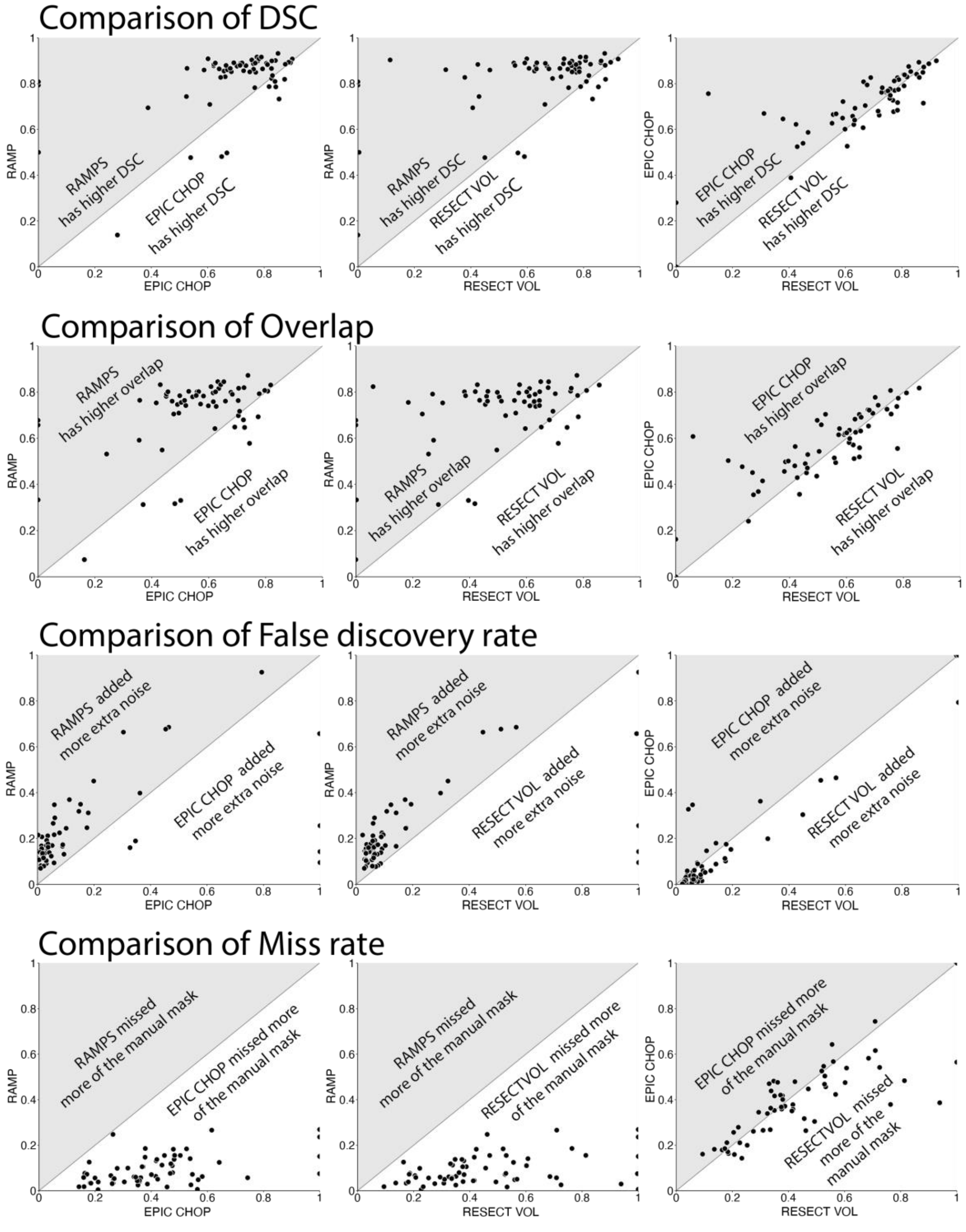

Figure S2: Comparison between pipelines. *Examination of the pipelines performance against one another under dice similarity coefficient (DSC), overlap, false discovery rate (FDR) and miss rate.*

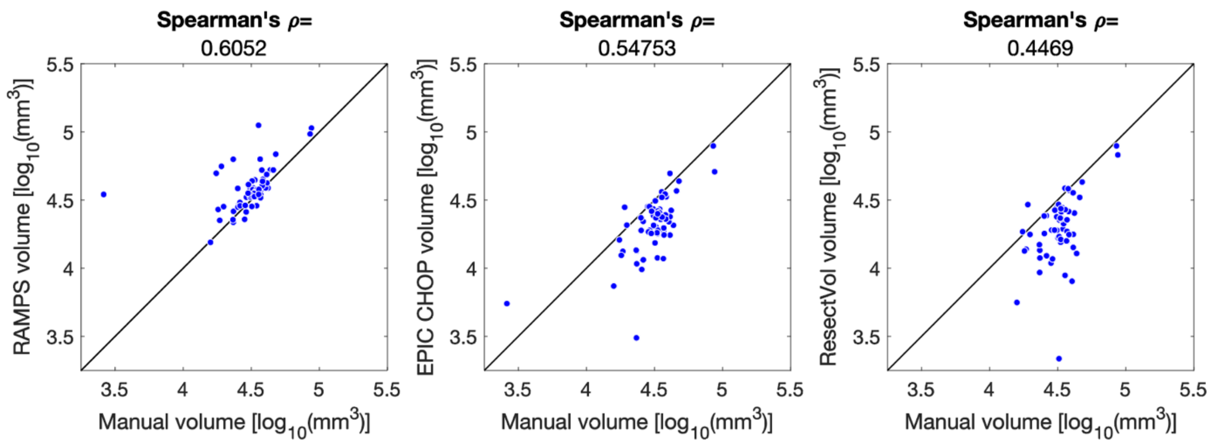

Figure S3: RAMPS achieves the highest correlation with manually delineated masks *(Spearman's rho 0.61), followed by Epic-CHOP with 0.55 and then ResectVol with a spearman's p of 0.45. This highlights that RAMPS produces mask of more appropriate size volumetric size whereas EPIC-Chop and ResectVol produce smaller volume masks.*

However, we strongly caution against relying solely on this approach when evaluating the performance of the three pipelines, as it provides no comment on the pipeline's ability to accurately delineate the resection cavity. Two masks could share identical volumes but be in entirely different regions and with differing shapes, thus resulting in completed failure to delineate the cavity (DSC = 0 and overlap = 0%).

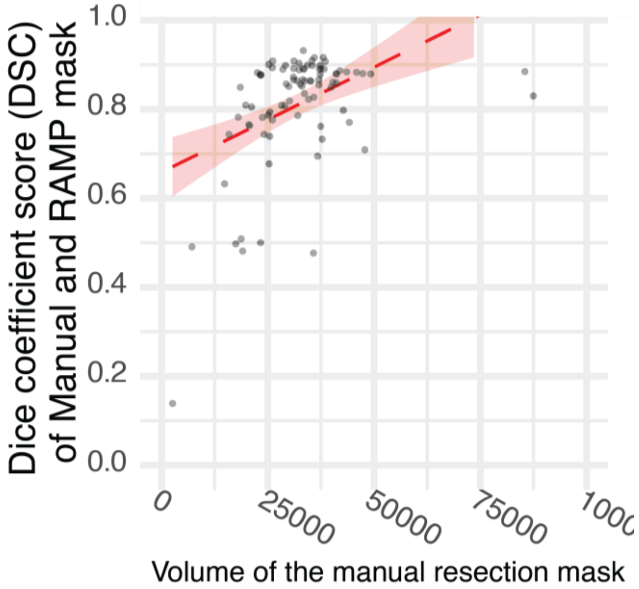

Figure S4: Relationship between manual mask volume and Dice Similarity Coefficient. *Spearman's correlation (0.39) reflects a relationship between the volume of the manually drawn resection mask and the Dice coefficients between the manual and RAMPS mask.*

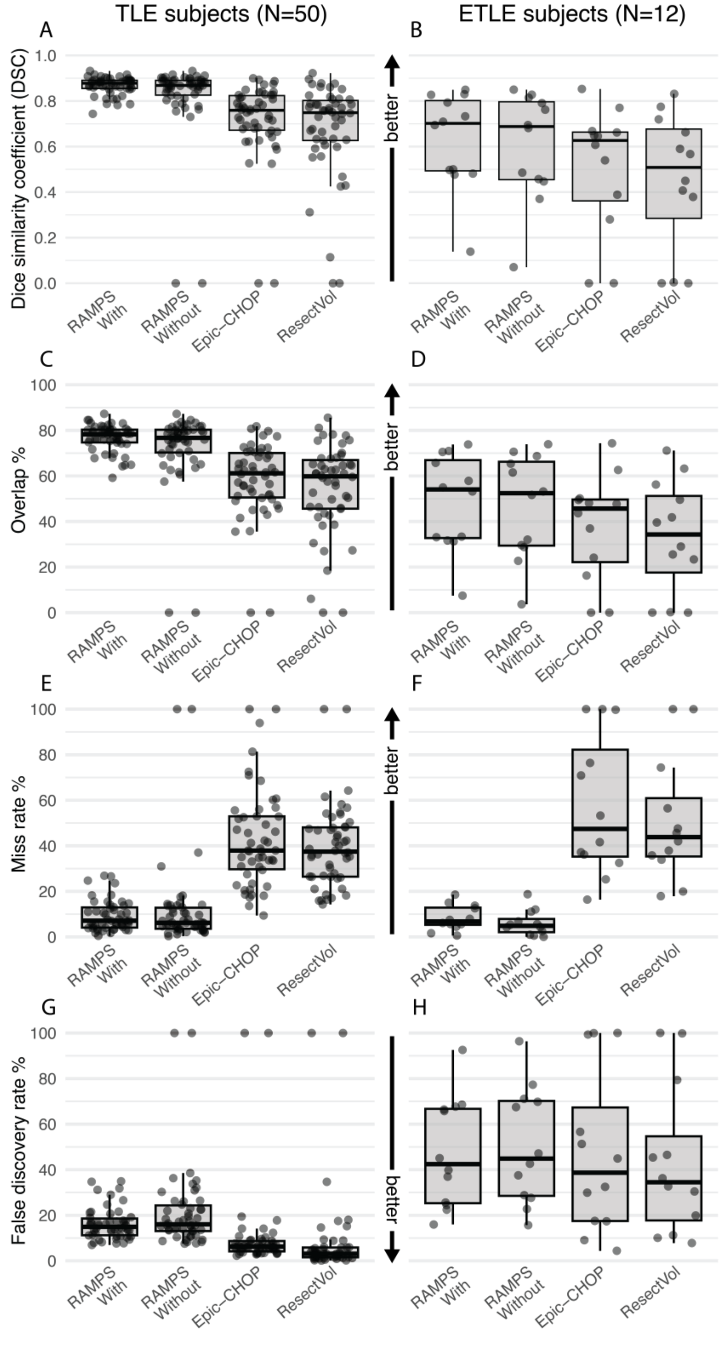

Figure S5: Pipeline comparison across cohorts. *The automated masks produced by RAMPS with lobe and hemisphere information, RAMPS without information, Epic-CHOP and ResectVol compared against a cohort of manual masks (N=62). The automated masks produced by RAMPS, Epic-CHOP and ResectVol compared against a cohort of manual masks in our cohort of TLE (N=50) and ETLE (N=12) cases. Each data point represents an individual patient.*

Table S1: Patient information used in the RAMPs comparison cohort. *Pathology are HS = Hippocampal sclerosis, FCD = Focal Cortical Dysplasia, DUAL = co-occurrence of two distinct pathologies, DNT = Dysembryoplastic neuroepithelial tumor, CAV = cavernoma, GL = Gliosis, Other = another pathology type, Unknown = Unknown pathology.* ***Type of resection*** *are Lx = lobectomy and Lesx = lesionectomy.*

Patient information		
N = 87	*Number in group*	*Percentage (2.dp)*
Hemisphere: *Left / Right*	*44 /43*	*50.6% / 49.4%*
Lobe: *Temporal / Frontal / Other ETLE*	*70 / 13 / 4*	*80.5% / 14.9 % / 4.6 %*
Pathology: *HS / FCD / DUAL / DNT / CAV / GL / Other / Unknown*	*35 / 9 / 5 / 5 / 2 / 1 / 12 / 18*	*40.2% / 10% / 5.7% / 5.7% / 2.3% / 1.1% / 13.8% / 20.7%*
Type of resection : *T Lx / F Lx / T Lesx / F Lesx / O Lx / OP Lx*	*67 / 13 / 3 / 2 / 1 / 1*	*77% / 14.9% / 3.5% / 2.3% / 1.1% / 1.1%*

Table S2: MRI acquisition parameters of scans used in the study. *All scans used in this study were acquired at 3 Tesla. Pre/Post operative voxel sizes are the voxel dimensions extracted from the header file, Other refers to voxel dimensions that only occur at maximum twice and can vary from close to the most common (0.94-0.94-1.2) to extremely varied (0.86-0.86-5.0).*

MRI specifications		
N = 87	*Number in group*	*Percentage (2.dp)*
Tesla: 3	*87*	*100%*
Pre operative voxel size to 2.d.p: 0.94-0.94-1.1 / 0.94-0.94-1.5 / 1.09-1.09-1.1 / 1.0-1.0-1.0 / Other	*47 / 10 / 8 / 8 / 14*	*54% / 11.5% / 9.2% / 9.2% / 16.1%*
Post operative voxel size to 2.d.p: 0.94-0.94-1.1 / 1.0-1.0-1.0 / 1.05-1.05-1.2 / Other	*51 / 7 / 7 / 22*	*58.6% / 8% / 8% / 25.3%*

Table S3: Comparison between Pipelines using a paired Wilcoxon signed-rank test. One-tailed tests were conducted in both directions to assess whether one pipeline outperformed the other across each metric, with the 'greater' alternative applied to DSC and overlap, and the 'less' alternative applied to miss rate and false discovery (FDR). For each metric, pipelines are ranked based on how close the median achieved is to the optimal score. **(Bold underscored indicates * $p < 0.05$).**

All patients (N = 62)						
Metric	Pipeline 1		Pipeline 2		Wilcoxon signed-rank test (paired)	
	Rank	Name	Rank	Name	Pipeline 1 Is better than Pipeline 2	Pipeline 2 Is better than Pipeline 1
DSC	1	RAMPS with info	2	RAMPS without info	6.272e-06	1
			3	Epic-CHOP	1.787e-08	1
			4	ResectVol	5.554e-10	1
	2	RAMPS without info	3	Epic-CHOP	9.482e-06	1
			4	ResectVol	3.583e-07	1
	3	Epic-CHOP	4	ResectVol	0.00348	0.9966
overlap	1	RAMPS with info	2	RAMPS without info	5.882e-06	1
			3	Epic-CHOP	3.083e-09	1
			4	ResectVol	3.906e-10	1
	2	RAMPS without info	3	Epic-CHOP	1.731e-06	1
			4	ResectVol	2.074e-07	1
	3	Epic-CHOP	4	ResectVol	0.003988	0.9961
miss rate	1	RAMPS without info	2	RAMPS with info	0.0002219	0.9998
			3	Epic-CHOP	5.554e-10	1
			4	ResectVol	3.129e-10	1
	2	RAMPS with info	3	Epic-CHOP	3.883e-12	1
			4	ResectVol	3.883e-12	1
	3	Epic-CHOP	4	ResectVol	0.02883	0.9717
FDR	1	Epic-CHOP	2	ResectVol	2.185e-06	1
			3	RAMPS with info	2.864e-06	1
			4	RAMPS without info	7.832e-07	1
	2	ResectVol	3	RAMPS with info	2.99e-07	1
			4	RAMPS without info	1.188e-07	1
	3	RAMPS with info	4	RAMPS without info	4.585e-08	1

Table S4: Metric comparison of each pipeline across the TLE and ETLE cohorts.

Median (IQR)								
	TLE (N = 50)				ETLE (N = 12)			
	RAMPS with	RAMPS without	Epic-CHOP	ResectVol	RAMPS with	RAMPS without	Epic-CHOP	ResectVol
DSC	88%(4%)	87% (7%)	76%(15%)	74%(18%)	70%(30%)	68%(34%)	62%(30%)	51%(40%)
overlap	78%(6%)	77% (10%)	61%(20%)	60%(21%)	54%(34%)	52%(37%)	46%(28%)	34%(34%)
miss rate	7%(9%)	6% (9%)	38%(22%)	38%(24%)	7%(8%)	5%(6%)	44%(26%)	47%(47%)
FDR	15%(7%)	16% (11%)	3%(4%)	6%(5%)	42%(41%)	45%(42%)	35%(37%)	38%(50%)

Table S5: Comparison between Pipelines TLE masks using a paired Wilcoxon signed-rank test. *One-tailed tests were conducted in both directions to assess whether one pipeline outperformed the other across each metric, with the 'greater' alternative applied to DSC and overlap, and the 'less' alternative applied to miss rate and false discovery rate (FDR). For each metric, pipelines are ranked based on how close the median achieved is to the optimal score.* ***(<u>Bold underscored indicates * p < 0.05</u>).***

TLE (N = 50)						
Metric	Pipeline 1		Pipeline 2		Wilcoxon signed-rank test (paired)	
	Rank	Name	Rank	Name	Pipeline 1 Is better than Pipeline 2	Pipeline 2 Is better than Pipeline 1
DSC	1	RAMPS with info	2	RAMPS without info	***<u>0.0001049</u>***	0.9999
			3	Epic-CHOP	***<u>4.903e-09</u>***	1
			4	ResectVol	***<u>3.1e-09</u>***	1
	2	RAMPS without info	3	Epic-CHOP	***<u>1.637e-06</u>***	1
			4	ResectVol	***<u>9.725e-07</u>***	1
	3	Epic-CHOP	4	ResectVol	***<u>0.009289</u>***	0.991
overlap	1	RAMPS with info	2	RAMPS without info	***<u>0.000101</u>***	0.9999
			3	Epic-CHOP	***<u>4.903e-09</u>***	1
			4	ResectVol	***<u>2.926e-09</u>***	1
	2	RAMPS without info	3	Epic-CHOP	***<u>1.49e-06</u>***	1
			4	ResectVol	***<u>8.836e-07</u>***	1
	3	Epic-CHOP	4	ResectVol	***<u>0.01124</u>***	0.9891
miss rate	1	RAMPS without info	2	RAMPS with info	***<u>0.006916</u>***	0.9933
			3	Epic-CHOP	***<u>6.447e-08</u>***	1
			4	ResectVol	***<u>3.789e-08</u>***	1
	2	RAMPS with info	3	Epic-CHOP	***<u>3.895e-10</u>***	1
			4	ResectVol	***<u>3.895e-10</u>***	1
	3	Epic-CHOP	4	ResectVol	***<u>0.04779</u>***	0.9532
FDR	1	Epic-CHOP	2	ResectVol	***<u>2.569e-06</u>***	1
			3	RAMPS with info	***<u>7.287e-07</u>***	1
			4	RAMPS without info	***<u>2.461e-07</u>***	1
	2	ResectVol	3	RAMPS with info	***<u>1.032e-07</u>***	1
			4	RAMPS without info	***<u>8.381e-08</u>***	1
	3	RAMPS with info	4	RAMPS without info	***<u>5.365e-06</u>***	1

Table S6: Comparison between Pipelines ETLE masks using a paired Wilcoxon signed-rank test. *One-tailed tests were conducted in both directions to assess whether one pipeline outperformed the other across each metric, with the 'greater' alternative applied to DSC and overlap, and the 'less' alternative applied to miss rate and false discovery rate (FDR). For each metric, pipelines are ranked based on how close the median achieved is to the optimal score.* ***(Bold underscored indicates * p < 0.05).***

ETLE (N = 12)						
Metric	Pipeline 1		Pipeline 2		Wilcoxon signed-rank test (paired)	
	Rank	Name	Rank	Name	Pipeline 1 Is better than Pipeline 2	Pipeline 2 Is better than Pipeline 1
DSC	1	RAMPS with info	2	RAMPS without info	***0.01709***	0.9866
			3	Epic-CHOP	0.1506	0.8669
			4	ResectVol	***0.02612***	0.9788
	2	RAMPS without info	3	Epic-CHOP	0.311	0.7153
			4	ResectVol	0.0647	0.9451
	3	Epic-CHOP	4	ResectVol	0.08408	0.9288
overlap	1	RAMPS with info	2	RAMPS without info	***0.02124***	0.9829
			3	Epic-CHOP	0.08813	0.9243
			4	ResectVol	***0.03198***	0.9739
	2	RAMPS without info	3	Epic-CHOP	0.1506	0.8669
			4	ResectVol	0.0647	0.9451
	3	Epic-CHOP	4	ResectVol	0.09866	0.9159
miss rate	1	RAMPS without info	2	RAMPS with info	***0.0007324***	0.9995
			3	Epic-CHOP	***0.0002441***	1
			4	ResectVol	***0.0002441***	1
	2	RAMPS with info	3	Epic-CHOP	***0.0002441***	1
			4	ResectVol	***0.0002441***	1
	3	Epic-CHOP	4	ResectVol	0.2249	0.8008
FDR	1	Epic-CHOP	2	ResectVol	0.08408	0.9288
			3	RAMPS with info	0.1902	0.8303
			4	RAMPS without info	0.1697	0.8494
	2	ResectVol	3	RAMPS with info	0.1506	0.8669
			4	RAMPS without info	0.1167	0.8982
	3	RAMPS with info	4	RAMPS without info	***0.0007324***	0.9995
